# Rank-based learning: a novel high-throughput algorithm resilient to missing data and effective for datasets with small sample size

**DOI:** 10.1093/bib/bbaf666

**Published:** 2025-12-12

**Authors:** Lulu Song, Hamid Khoshfekr Rudsari, Johannes F Fahrmann, Jody Vykoukal, Sam Hanash, James P Long, Kim-Anh Do, Ehsan Irajizad

**Affiliations:** Department of Biostatistics, University of Texas MD Anderson Cancer Center, Houston, TX, USA; Department of Biostatistics, University of Texas MD Anderson Cancer Center, Houston, TX, USA; Department of Cancer Prevention, University of Texas MD Anderson Cancer Center, Houston, TX, USA; Department of Cancer Prevention, University of Texas MD Anderson Cancer Center, Houston, TX, USA; Department of Cancer Prevention, University of Texas MD Anderson Cancer Center, Houston, TX, USA; Department of Biostatistics, University of Texas MD Anderson Cancer Center, Houston, TX, USA; Department of Biostatistics, University of Texas MD Anderson Cancer Center, Houston, TX, USA; Department of Biostatistics, University of Texas MD Anderson Cancer Center, Houston, TX, USA

**Keywords:** rank-based learning, high-throughput omics, missing data, machine learning

## Abstract

High-throughput omics data present challenges for binary classification due to platform variability, batch effects, missing values, and high dimensionality. This study presents a novel Rank-Based Learning (RBL) method that leverages relative feature rankings to improve robustness and generalizability. We evaluated RBL against established methods like Logistic Regression (LR) and Random Forest (RF) using simulated data and two real-world plasma proteomics datasets: early-stage small cell lung cancer (SCLC) and duodenopancreatic neuroendocrine tumors (dpNET) in patients with Multiple Endocrine Neoplasia type 1 (MEN1). In simulation experiments, RBL outperformed LR under conditions involving batch effects, missing data, and varying numbers of true differential features. In SCLC, RBL yielded a test AUC of 0.76 (95% CI: 0.42–1.00), surpassing LR with Lasso (0.65 [95% CI: 0.47–0.84]) and RF with feature importance (0.59 [95% CI: 0.33–0.87]). In dpNET, RBL achieved an AUC of 0.83 (95% CI: 0.67–0.97) on the development set and 0.80 (95% CI: 0.54–0.98) on the test set, outperforming LR with Lasso (0.57 [95% CI: 0.40–0.77]) and RF with feature importance (0.53 [95% CI: 0.29–0.77]). By emphasizing feature ranking rather than absolute expression levels, RBL effectively mitigates the impact of non-biological variation. Overall, RBL improves the predictive accuracy of diagnostic models for complex diseases and provides a promising framework for developing more reliable and generalizable diagnostic tools from omics data, moving them closer to clinical application.

## Introduction

High-throughput omics technologies, such as genomics, proteomics, and metabolomics, have revolutionized biomedical research by enabling comprehensive analysis of biological molecules on a large scale [1–4]. These technologies facilitate the identification of genetic variants, protein expression patterns, and metabolic profiles, offering critical insights into the molecular mechanisms underlying diseases. By capturing vast quantities of biological information, omics technologies have substantially advanced our ability to classify disease subtypes and identify biomarkers for early detection, prognosis, and therapeutic response. For example, we conducted a comprehensive proteomic profiling of plasma samples from small-cell lung cancer (SCLC) patients and identified circulating protein markers associated with disease pathogenesis [5]. Similarly, gene expression profiling was used to classify breast cancer subtypes and predict clinical outcomes, demonstrating the prognostic power of genomic data [6]. More recent studies have further enhanced cancer subtype classification and biomarker discovery through multi-omics integration and deep learning approaches [7–9].

Despite these advances, high-throughput omics technologies face key limitations due to their dependence on specific experimental platforms [10–13]. Many studies are constrained by the technical characteristics of the platforms used to generate the data, which limits the generalizability of the findings to be cross validated across different platforms. Most existing models rely on the absolute magnitudes of selected features within a particular platform [14, 15]. Consequently, variations in the data pipeline, such as differences in sample handling protocols, can introduce systematic shifts that lead to inconsistent predictions when the same algorithm is applied to new data. Additionally, such algorithms often depend on the platform’s sensitivity to reproducibly capture the same features. Consequently, when applied to data generated from a different platform, their performance may degrade, resulting in poor reproducibility across studies or experimental conditions [15–17].

Another common limitation in omics research is the "large p, small n" problem, in which the number of features (p) greatly exceeds the number of samples (n) [18–21]. This challenge is typically mitigated by applying feature-selection procedures to retain the most discriminative variables across experimental conditions [22–27]. However, this process can inadvertently limit the discovery potential of omics studies, as it focuses on a small subset of features, and may exclude biologically relevant signals.

Recent deep learning frameworks, such as scAMZI, scRGCL, and scMGATGRN, have advanced omics and single-cell analyses by applying attention mechanisms and graph neural networks to capture complex biological dependencies [28–30]. Similarly, ensemble transfer learning models, such as the voting transfer approach, have improved protein-level classification under high-dimensional and imbalanced conditions. While these deep learning methods demonstrate strong predictive performance, they often require large datasets and complex architectures that are often prone to overfitting, particularly in small-sample high-dimensional settings. [31, 32].

To address these challenges, we developed a novel Rank-Based Learning (RBL) algorithm designed that enhances robustness to platform variability and data inconsistencies. Instead of relying on absolute values, RBL leverages the ranking distribution of features for classification, identifying the optimal ranking patterns that that optimally distinguish between sample classes across multiple datasets. This approach preserves the full spectrum of informative features, reducing the risk of excluding potentially important signals. Because it operates on rankings rather than raw measurements, RBL facilitates cross-platform validation and remains resilient to missing values by incorporating all available features within each sample.

This paper is organized as follows. Section 2 presents the superiority of RBL method over LR in simulations and over both LR and RF in real-world datasets. Section 3 provides a discussion of our findings and concluding remarks. Section 4 describes the RBL framework.

## Results

We evaluated the performance of the RBL method in four different simulation studies and two real-world applications as explained in detail in the Material and Method section.

### Simulation studies

#### True differential scenario

In simulations with 60 observations (30 cases and 30 controls) and 300 features, for 1% true differential features, the test AUCs were 0.82 (95% CI: 0.80–0.84) for RBL and 0.83 (95% CI: 0.80–0.87) for LR. As the proportion of true differential features increased to 5%, 10%, and 20%, RBL’s test AUCs improved to 0.95 (95% CI: 0.94–0.96), 0.99 (95% CI: 0.99–1.00), and 1.00 (95% CI: 1.00–1.00), respectively. In comparison, LR achieved test AUCs of 0.88 (95% CI: 0.86–0.91), 0.85 (95% CI: 0.83–0.88), and 0.85 (95% CI: 0.83–0.88) for the same scenarios (Supplementary Table S1; Supplementary Fig. S1).

In simulations with an increased sample size of 200 observations (100 cases and 100 controls), RBL consistently outperformed LR in scenarios with 5% true differential features, achieving a test AUC of 0.90 (95% CI: 0.89–0.91) compared to LR’s 0.85 (95% CI: 0.83–0.86) (Supplementary Table S2).

#### Missing scenario

With 1% true differential features and 10% missing values, RBL achieved a test AUC of

0.81 (95% CI: 0.79–0.83), compared to 0.69 (95% CI: 0.66–0.73) for LR. As the percentage of missing values increased from 10% to 50%, RBL consistently outperformed LR in both development and test set. Specifically, at 50% missingness, RBL attained a test AUC of 0.72 (95% CI: 0.69–0.74) versus 0.53 (95% CI: 0.51–0.56) for LR. This trend continued at higher proportions of true differential features. At 5% true differential features and 50% missingness, RBL maintained a test AUC of 0.82 (95% CI: 0.80–0.84), whereas LR had a test AUC of 0.55 (95% CI: 0.52–0.58). At 10% true differential features and the same level of missingness, RBL’s test AUC was 0.92 (95% CI: 0.90–0.94) compared to LR’s AUC of 0.59 (95% CI: 0.56–0.61). Finally, at the highest true differential feature level (20%) and 50% missingness, RBL achieved a test AUC of 0.95 (95% CI: 0.94–0.97), substantially outperforming LR’s AUC of 0.63 (95% CI: 0.60–0.66) (Supplementary Table S3; Supplementary Fig. S2).

#### Batch effect scenario

With 1% true differential features, RBL achieved a test AUC of 0.98 (95% CI: 0.98–0.98), outperforming LR at 0.69 (95% CI: 0.67–0.70). At 5%, the test AUC for RBL reached 1.00 (95% CI: 1.00–1.00), while LR attained 0.70 (95% CI: 0.69–0.72). At 10% and 20% true differential features, RBL consistently achieved test AUCs of 1.00 (95% CI: 1.00–1.00), whereas LR reached test AUCs of 0.72 (95% CI: 0.70–0.73) and 0.71 (95% CI: 0.70–0.72), respectively (Supplementary Table S4; Supplementary Fig. S3).

#### Correlation scenario

In this setting, both methods showed consistently high development AUCs. RBL test AUCs increased with the proportion of true differential features, from 0.86 (95% CI: 0.83–0.88) at 1% to 1.00 (95% CI: 1.00–1.00) at 10% and 20%. LR followed a similar trend, with test AUCs rising from 0.96 (95% CI: 0.94–0.98) to 1.00 (95% CI: 1.00–1.00). Both methods performed equally well when 5% or more of the features were truly differential. (Supplementary Table S5; Supplementary Fig. S4).

### Real-world data application

#### Proteomics dataset for detection of newly diagnosed early-stage small-cell lung cancer

The dataset contains 4,388 gene-protein products. Among the cases, eight are male and seven are female, with the same distribution in the controls. Six cases are in stage I, and nine are in stage II. The mean ages for cases and controls are 67 and 64 years, respectively (Table 1).

**Table 1:** Patient and tumor characteristics for SCLC cohort

Patient and Tumor Characteristics	cases	controls
N	15	15
Age, mean (std)	67 (10)	64 (5)
Sex, N (%)		
Male	8 (53.3%)	8 (53.3%)
Female	7 (46.7%)	7 (46.7%)
Stage, N (%)		
I	6 (40%)	-
II	9 (60%)	-
Smoking Pack years, mean (std)	63 (27)	51 (18)

RBL achieved an AUC of 0.84 (95% CI: 0.60–1.00) on the development set and 0.76 (95% CI: 0.42–1.00) on the test set. In comparison, LR and RF with seven features after forward feature selection yielded AUCs of 0.98 (95% CI: 0.92–1.00) and 1.00 (95% CI: 0.98–1.00) on the development set, 0.57 (95% CI: 0.49– 0.88) and 0.57 (0.43–0.93) on the test set, respectively. LR with six features selected by Lasso achieved an AUC of 0.95 (95% CI: 0.90–1.00) on the development set and 0.65 (95% CI: 0.47–0.84) on the test set. Using 20 RankProd-selected features, LR achieved **1.00 (95% CI: 0.90–1.00)** on the development set and **0.59 (95% CI: 0.37–0.92)** on the test set. RF with two features selected by feature importance showed an AUC of 1.00 (95% CI: 0.98–1.00) and 0.59 (95% CI: 0.36–0.87) on the development and test set, respectively (Table 2; Supplementary Fig. S5).

**Table 2:** Performances of RBL, LR, and RF in plasma proteomics for detection of early-stage SCLC

**Model**	**Feature Selection**	**Development set AUC (95% CI)**	**Test set AUC (95% CI)**
**LR**	Lasso	0.95 (0.90–1.00)	0.65 (0.47–0.84)
**LR**	Rank Product	1.00 (0.90–1.00)	0.59 (0.37–0.92)
**LR**	Forward selection	0.98 (0.92–1.00)	0.57 (0.49–0.88)
**RF**	Feature importance	1.00 (0.98–1.00)	0.59 (0.36–0.87)
**RF**	Forward selection	1.00 (0.98–1.00)	0.57 (0.43–0.93)
**RBL**	—	0.84 (0.60–1.00)	0.76 (0.42–1.00)

#### Proteomics dataset for detection of duodenopancreatic neuroendocrine tumors in patients with multiple endocrine neoplasia type 1

The dataset contains 10,937 gene-protein products. Among the cases, six are male and eight are female. Control group 1 consists of 13 male and 15 female. Control group2 includes seven male and seven female (Table 3).

**Table 3:** Patient and tumor characteristics for the MEN1 cohort

	Cases	Controls#1	Controls#2
n	14	28	14
Sex, n (%)			
Male	6 (43)	13 (46)	7 (50)
Female	8 (57)	15 (54)	7 (50)
Age (median, IQR)	52.5 (41.8-60)	39.5 (28.5-58)	29.5 (22-38.5)
BMI (median, IQR)^a^	26 (22.8-32.3)	26 (23-36)	23 (20.5-24.5)
Collection site, n (%)			
MDACC	5 (36)	3 (11)	1 (7)
NIH-NIDDK	5 (36)	0 (0)	1 (7)
UMCU	4 (29)	25 (89)	12 (86)
PanNET	13^b^	28	—
Prior dpNET surgery, n (%)	7 (50)	1 (4)	—
Size largest PanNET resected			
≤20 mm	2	—	—
>20 mm	3	1	—
N/A (only duodenal/lymph node)	2	—	—
Size of largest PanNET, n (%) (in situ at sample collection)	12 (4-29)	11 (6-23)	—
<20 mm	11 (79)	26 (93)	—
≥20 mm	2 (14)	2 (7)	—

Abbreviations: BMI, body mass index; dpNET, duodenopancreatic neuroendocrine tumor; IQR, interquartile range; MDACC, MD Anderson Cancer Center; N/A, not applicable; NIH-NIDDK, National Institutes of Health-National Institute of Diabetes and Digestive and Kidney Diseases; PanNET, pancreatic neuroendocrine tumor; UMCU, University Medical Center Utrecht.

^*^a^*^BMI data were not available for 10 control subjects.

^*^b^*^One case had total or partial pancreatectomy but presented with dpNET-related liver metastasis at the time of blood collection.

RBL yielded an AUC of 0.83 (95% CI: 0.67–0.97) on the development set and 0.80 (95% CI: 0.54–0.98) on the test set. In comparison, LR with 28 Lasso-selected features yielded a development AUC of 0.80 (95% CI: 0.56–0.80) and a test AUC of 0.57 (95% CI: 0.40–0.77). LR with two RankProd-selected features achieved development and test AUCs of 0.74 (95% CI: 0.50-0.88) and 0.50 (95% CI: 0.18, 0.85), respectively. RF with four features selected by feature importance demonstrated a development AUC of 1.00 (95% CI: 0.96–1.00) and a test AUC of 0.53 (95% CI: 0.29–0.77) (Table 4; Supplementary Fig. S6).

**Table 4:** Performances of RBL, LR, and RF in plasma proteomics for detection of dpNET in patients with MEN1

**Model**	**Feature Selection**	**Development set AUC (95% CI)**	**Test set AUC (95% CI)**
**LR**	Lasso	0.80 (0.56-0.80)	0.57 (0.40-0.77)
**LR**	Rank Product	0.74 (0.50, 0.88)	0.50 (0.18, 0.85)
**RF**	Feature importance	1.00 (0.96-1.00)	0.53 (0.29-0.77)
**RBL**	—	0.83 (0.67-0.97)	0.80 (0.54-0.98)

## Discussion

This study presents a novel machine learning method, termed Rank-Based Learning (RBL), designed to classify high-throughput omics data by estimating the optimal ranking profile of features that best distinguishes two groups, such as cancer versus healthy controls. The RBL employs a similarity-based scoring function to assess rankings and uses the Metropolis–Hastings stochastic search algorithm to identify the optimal feature order. The method was systematically evaluated across four simulation scenarios as well as two real-world, SCLC and duodenopancreatic neuroendocrine tumors (dpNET), and compared with established approaches including Logistic Regression (LR) and Random Forest (RF). RBL's performance improved with increasing proportions of true differential features and demonstrated robustness under conditions of missingness and batch effects. Notably, RBL outperformed LR and RF in test AUC across both real-world datasets.

By leveraging **feature rankings rather than absolute measurements**, RBL effectively handles high-dimensional data and enhances biomarker discovery when the sample size is limited relative to the number of features. This property makes RBL particularly well-suited for omics data, which are often affected by technical and biological variability [10]. Through its reliance on relative orderings, RBL mitigates the impact of batch effects and normalization discrepancies that commonly challenge high-throughput analyses. This robustness enhances its applicability in clinical research settings, where data heterogeneity is a significant challenge. Additionally, RBL incorporates all available quantified features that makes it more generalizable to new test datasets compared to methods that rely on feature selection, improving generalizability to independent test datasets and maintaining stability even in the presence of missing values.

In both SCLC and dpNET datasets, RBL exhibited slightly lower development AUCs compared to conventional methods but **consistently superior generalization** on test data. Specifically, RBL achieved development AUCs of 0.84 (95% CI: 0.60–1.00) for SCLC and 0.83 (95% CI: 0.67–0.97) for dpNET. This difference likely reflects RBL's reliance on relative feature rankings rather than dataset-specific absolute intensities. Despite this, RBL’s test set AUCs: 0.76 (95% CI: 0.42–1.00) for SCLC and 0.80 (95% CI: 0.54–0.98) for dpNET, underscoring its stronger generalization to unseen data.

In the SCLC dataset, RBL identified an optimal feature rank profile (Supplementary Table S7) that captures distinctive relative expression patterns characteristic of SCLC. Within this profile, several known SCLC-associated overexpressed proteins, including NCAM1, FUT1, KSR2 and TFRC [33–36], were consistently ranked above ACTB, a highly abundant and ubiquitously expressed protein often used as a stable internal control in molecular biology experiments due to its relatively constant expression across different cell types and physiological conditions [37]. This ranking pattern indicates that these SCLC-related proteins are consistently elevated relative to ACTB in SCLC cases, whereas such ordering is absent or reversed in controls.

Similarly, in the dpNET dataset, proteins such as IGFBP2, CHI3L1, TIMP1, and COL18A1 also consistently ranked above ACTB in the optimal feature rank profile (Supplementary Table S8). These proteins have previously been reported as elevated in early-stage pancreatic ductal adenocarcinoma (PDAC) cases compared to healthy or benign conditions [38, 39], further demonstrating RBL's capability to uncover biologically meaningful relative expression patterns across different cancer types.

Beyond these findings, biological validation of the learned ranking profiles can further strengthen the interpretability. The high-ranking proteins identified by RBL can be validated through independent experimental assays, such as ELISA, Western blot, or immunohistochemistry, to confirm their differential abundance between cancer and control samples. Moreover, proteins that consistently appear above stable housekeeping proteins (e.g., ACTB) in the ranking profile may represent biologically relevant targets. These candidates can be investigated using functional studies, such as gene knockdown, knockout, or overexpression experiments, to determine their mechanistic roles in tumor development or progression. Together, these approaches may provide a biologically grounded framework for validating and interpreting RBL-derived rank profiles across independent datasets.

This study has several limitations. First, RBL is designed for binary classification and cannot be directly applied to multi-class problems. However, the framework has flexibility to be extended by adapting its loss function to evaluate similarity across multiple classes. One approach is to learn pairwise rank profiles between classes using one-vs-one or one-vs-rest schemes, where the total similarity score, originally computed as the difference between cases and controls, is redefined to compare each target class against its reference class (e.g., all remaining samples). Future work may also explore a unified formulation that learns a single ranking profile to maximize separation among class-specific mean similarity scores while minimizing within-class variability. These extensions would enable RBL to generalize naturally to multi-class settings while preserving its rank-based framework.

Second, RBL focuses exclusively on ranking features without considering the magnitude of intensity differences, potentially overlooking certain quantitative effects.

Furthermore, RBL can be computationally intensive, especially for datasets with large number of features. To improve efficiency, future implementations could leverage **Numba-based just-in-time (JIT) compilation, which** compiles **Python functions into** optimized **machine code at runtime; delta-based incremental updates** that reuses pairwise comparisons during MCMC iterations**; and parallelization across high-performance computing (HPC) clusters** to further accelerate computation.

Additionally, while RBL mitigates missing data by excluding pairs with missing values, RBL is appropriate when missingness is random, it may introduce bias if missingness is non-random. This limitation is not unique to RBL; existing methods, including statistical and machine learning approaches, also struggle with missing-not-at-random (MNAR) mechanisms [40–42]. Addressing such informative missingness remains a well-recognized challenge in omics analyses and represents an important direction for future methodological development.

In conclusion, RBL is a promising learning algorithm for binary classifications with high-throughput omics data as the inputs, even in the presence of missing data and batch effects.

## Materials and Methods

### Development of RBL

Let $A$ be a permutation of $\left\{1,\dots, p\right\}$, which we call a feature rank profile. Using a training data set, we seek a feature rank profile $A$ which is highly concordant with group 1 (i.e. cases) but discordant with group 2 (i.e. controls) when comparing two groups of data, defined precisely in the following paragraphs. Once the feature rank profile is determined, it can be used to compute risk scores for a test set.

Let $x\in{R}^p$ denote a vector of biomarker values (e.g. protein expression). We define a similarity score to indicate if the ranking of features $i$ and $j$ in $x$ agrees with rank profile $A$. Specifically let


(1) \begin{equation*}\! u{\left(x,A\right)}_{ij}=\left\{\begin{array}{c}1,\mathrm{if}\ \left({A}_i-{A}_j\right)\left({x}_i-{x}_j\right)>0\\{}-1,\mathrm{if}\ \left({A}_i-{A}_j\right)\left({x}_i-{x}_j\right)<0\\{}\!\!0,\mathrm{if}\ \left({A}_i-{A}_j\right)\left({x}_i-{x}_j\right)=0\ \mathrm{or}\ {x}_i\,\,\,\mathrm{or}\ {x}_j\ \mathrm{is}\ \mathrm{missing}\end{array}\right. \end{equation*}

for $i,j\in \left\{1,\dots, p\right\}.$ If the relative rank between $i$ and $j$ is the same in both rank profile $A$ and feature vector $x$, the score is 1. If the ordering between them is reversed, the score is −1. If either ${x}_i$ or ${x}_j$ is missing, the ordering cannot be determined, and the score is 0.

We sum the scores over all possible pairs of features to obtain the similarity score of $x$ with $A$:


(2) \begin{equation*} U\left(x,A\right)={\sum}_{i,j\in \left\{1,\dots, p\right\}}u{\left(x,A\right)}_{ij}. \end{equation*}

Let ${x}_{1,k}\in{R}^p$ for $k=1,\dots, {n}_1$ denote biomarker expression vectors for ${n}_1$ cases and ${x}_{2,k}\in{R}^p$ for $k=1,\dots, {n}_2$ denote biomarker expression vectors for ${n}_2$ controls. We sum similarity across cases and controls separately and compute the difference. The result is termed the total similarity score (TSS):


$$ {U}_{cases}(A)=\frac{\sum_{k=1}^{n_1}U\left({x}_{1,k},A\right)}{n_1}, $$

$$ {U}_{controls}(A)=\frac{\sum_{k=1}^{n_2}U\left({x}_{2,k},A\right)}{n_2}, $$

(3) \begin{equation*} \mathrm{TSS}(A)={U}_{cases}(A)-{U}_{controls}(A). \end{equation*}

The goal is to find the ranking profile $A$ that maximizes the TSS:


(4) \begin{equation*} \hat{A}={\displaystyle \begin{array}{c} argmax\\{}A\end{array}}\ TSS(A). \end{equation*}

We discuss algorithms for solving Equation 4 below. Once $\hat{A}$ is determined, risk scores for each sample in the test set T are computed using Equation 2 by evaluating their similarity to the learned ranking profile.

### Steps repetition, pseudo-code, and flowchart of the RBL

**Figure 1:**
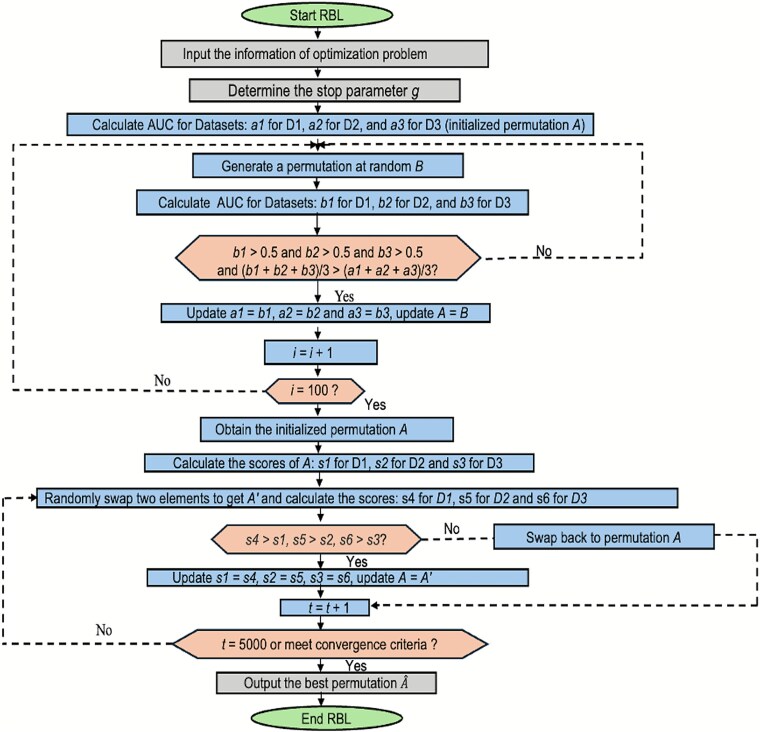
Flowchart of looking for the optimal permutation.

The objective function is defined over the space of all permutations of *p* elements, resulting in a factorially large, discrete search space. As traditional gradient-based methods are inapplicable, we adopt the Metropolis–Hastings stochastic search method [43], which is effective for exploring large discrete spaces. The process starts by initializing 100 random permutations and calculating their total similarity scores as described in Equations (1)–(3), from which corresponding AUCs are calculated. The permutation with the highest combined AUC across Development Data 1 (D1), D2, and D3 is selected as the initial candidate. At each iteration, two elements in the permutation are swapped, similarity scores are recalculated, and the permutation is updated if performance improves. If not, the swap is reverted, and a new pair is randomly selected. This process continues until no further improvement is observed in D1 based on a predefined convergence criterion. The best-performing permutation is then selected. A flowchart of the process is shown in Fig. 1, and the pseudo-code is provided in Table 5.

**Table 5:** The algorithm of finding the optimal similarity score

Algorithm 1. Pseudo-code of RBL
Start RBL Input the information of optimization problem. Determine the stop parameter *g*. Phase 1: Specialized Initialization Calculate AUCs for Datasets: *a1*​ for D1, *a2*​ for D2, and *a3*​ for D3 (initialized permutation *A*). For *i* = 1 to 100: Generate a permutation *B* at random, calculate similarity scores using Equation (3), and calculate AUCs: *b1* for D1, *b2*​ for D2, and *b3*​ for D3. If *b1* > 0.5 and *b2* > 0.5 and *b3* > 0.5 and (*b1* + *b2* + *b3*)/3 > (*a1* + *a2* + *a3*)/3: Update *a1* = *b1*, *a2* = *b2* and *a3* = *b3*, and update *A* = *B*. End for. Phase 2: Search for the Optimal Permutation Calculate the similarity scores of the initialized permutation *A*: *s1* for D1, *s2* for D2 and *s3* for D3 using Equation (3). Calculate the AUCs of the initialized permutation *A*: *auc_train_prev* for D1, *auc_val1_prev* for D2 and *auc_val2_prev* for D3. For *t* = 1 to 5000: Swap two elements *i* and *j* at random and get *A'*: *A[i]*, *A[j]* = *A[j]*, *A[i]*. Calculate similarity scores for *A'*: *s4* for D1, *s5* for D2 and *s6* for D3. Calculate AUCs for *A'*: *auc_train* for D1, *auc_val1* for D2 and *auc_val2* for D3. If *s4* > *s1*, *s5*>*s2* and *s6*>*s3*: Update *s1* = *s4*, *s2* = *s5*, *s3* = *s6*, and update *A* = *A'*. Update *auc_train_prev = auc_train, auc_val1_prev = auc_val1, auc_val2_prev = auc_val2.* Else: Revert the permutation back to *A*. Early Stopping Condition: **If** t > 0 **and** t (mod 500) = 0 **then:** Set *last_checkpoint_t* = *t*. Set *auc_at_last_checkpoint* = *auc_train_prev*. **If** t ≥ last_checkpoint_t + last_checkpoint_t × g **and** *auc_train_prev* ≤ *auc_at_last_checkpoint* **then**: Break from loop. End for. Output the best permutation: $\hat{A}$ End RBL

### Algorithm input and output

Algorithm 1 in Table 5 performs searching the optimal similarity score based on the following input and output:

Input: **Parameter**  *g*  **(**defining the convergence criterion by limiting the number of iterations) and **development datasets (D1, D2, D3 –** labeled datasets containing feature vectors and class labels, e.g., case or control),

Output: Optimal permutation $\hat{A}$= {*a*_1_, *a*_2_, ..., *a*_p_}, where each *a*_i_ ∈ $\left\{1,\dots, p\right\}$ denotes the index of the feature ranked at position *i*.

### Computational complexity analysis

RBL consists of three main components: initialization, score computation, and iterative permutation updates. Among them, *C* denotes the number of permutations generated for initialization, *N* denotes the number of samples, *K* denotes the number of feature pairs. *T* denotes the maximum number of iterations, and *P* denotes the number of parameters needed to be validated. The computation complexities of calculating similarity score, initialization and permutation updates are O(*N*×*K*)), O(*C*×*N*×*K*), and O(*T*×*N*×*K*), respectively. Therefore, the total complexity of RBL is O((*C*+*T*) ×*N*×*K*×*P*). Given this computational cost, RBL could be applied through high-performance computing (HPC) resources. Computational settings and runtime details are summarized in Supplementary Table S6.

### Model evaluation

For each test sample, a similarity score was computed by comparing its feature ranking to the optimal rank profile learned from the training data. Higher scores indicated greater alignment with case-like patterns; lower scores suggested control-like profiles. These similarity scores served as continuous prediction outputs. The performance of RBL was evaluated using the Area Under the Receiver Operating Characteristic Curve (AUC). We compared the performance of RBL with LR and RF, incorporating various feature selection strategies including LR with Lasso, LR with Rank Product-based feature selection, a non-parametric method that identifies features consistently ranked across samples [44], RF with feature importance-based selection, and forward feature selection in the real datasets. The performance of RBL is compared with LR with forward feature selection in simulation datasets. For forward selection, features were sequentially selected using LR, with the optimal feature set determined via cross-validation. The resulting feature subset was then used to train both LR and RF models. For the real datasets, confidence intervals (95% CI) for AUC were estimated via bootstrap (1,000 resamples with replacement). For simulation experiments, 95% CIs were computed using the t-distribution to assess mean AUC performance across simulation replicates (*n* = 100), calculated as:

*CI* = mean ± *t_α/2, df_* × *SE*,

where $\alpha$= 0.05, and t*_α/2, df_* is the critical value from the t-distribution with *df* = *n* − 1 degrees of freedom, and *SE* is the standard error.

### Software implementation

RBL was implemented in Python 3, and the source code is available at:

https://github.com/lulusong512/Rank-Based-Learning.git.

### Dataset description

#### Simulation studies

We evaluated the performance of RBL using simulated data, comparing it with LR with forward feature selection across various scenarios described below. Unless otherwise specified, all datasets contained 300 features and 60 observations (30 cases and 30 controls). The batch effect scenario included 120 observations (60 cases and 60 controls). To further assess consistency and robustness, we conducted supplementary simulations with an increased sample size (200 observations: 100 cases and 100 controls) on scenarios with 5% true differential features. Features were sampled from normal distributions with means and variances randomly chosen from uniform distributions of *U*(50, 70) and *U*(1, 80), respectively. The simulations were considered:

**a. Basic true differential scenarios**

To assess performance under varying signal strengths, we simulated datasets containing 1%, 5%, 10%, and 20% truly differentially expressed features. Differential expression was induced by adding a constant (sampled from *N* (60,60)) to the selected features in one class. Significance was verified via two-sample t-tests.

**b. Missing data scenarios**

To assess robustness to missingness, increasing proportions of data were randomly removed, from 10% up to 50%, which were introduced at random.

**c. Batch effect scenarios**

Batch effects—systematic variations arising from differences in processing time, operator, equipment, or experimental conditions—were introduced to examine model robustness.

We added batch-specific constants to all features within each group, altering absolute values while preserving within-sample rankings. Because RBL relies on feature ranks rather than magnitudes, it is expected to be robust to such shifts.

For the batch effect scenarios, the basic true differential dataset was divided into three groups of 20 observations each (10 cases and 10 controls). Batch effects, sampled from *U*(100, 300), *U* (300, 500), and *U*(500, 700) were added to the respective groups, increasing absolute intensities but preserving rank order.

**d. Correlation scenarios**

To evaluate model sensitivity to feature correlation, we induced high correlation among truly differential features by adding shared values from *N*(10,10) to the case group.

#### Model training and testing in the simulation datasets

For the basic differential, missing, and correlation scenarios, the datasets were split into 70% training and 30% test sets. For the batch effect scenario, models were trained on unshifted data and evaluated on batch-affected data (60 observations each).

#### Proteomics dataset for detection of newly diagnosed early-stage SCLC

The dataset consisted of plasma samples from 15 newly diagnosed early-stage SCLC cases and 15 matched controls (age, sex, and smoking status) from MD Anderson Cancer Center (MDACC). SCLC cases were obtained from the Genomics Marker-Guided Therapy Initiative (GEM-INI) project, while controls were selected from the Lung Cancer Early Detection Assessment of Risk and Prevention (LEAP) study (IRB protocol 2013-0609). All controls remained cancer-free for at least four years after blood collection. Additional information is provided elsewhere [5].

#### Proteomics dataset for detection of dpNET in patients with multiple endocrine neoplasia type 1

This proteomics dataset for dpNET detection in patients with Multiple Endocrine Neoplasia type 1 (MEN1) was collected through an international collaboration between MD Anderson Cancer Center (MDACC), the National Institutes of Health (NIH), and the University Medical Center Utrecht (UMCU). All biospecimens and associated retrospectively collected clinical data were approved under MDACC protocol PA19-0498, with a waiver of informed consent. EDTA plasma samples were obtained from 14 MEN1 case subjects presenting with liver metastases from a dpNET and two types of controls: patients with MEN1 and a nonmetastatic (distant or regional) indolent dpNET (n = 28; controls-1) defined by at least 3 years of follow-up after a dpNET diagnosis and imaging taken more than 1 year after blood draw confirming absence of distant or regional metastases and patients with MEN1 without a visible dpNET or other NET (n = 14; controls-2), confirmed negative for dpNETs at blood collection time by either combined conventional and somatostatin-receptor imaging, or conventional imaging taken ≥6 months post-blood draw. Patients were included if they met MEN1 diagnostic criteria: (i) a confirmed germline MEN1 mutation; (ii) one of the three major manifestations (parathyroid, pituitary, dpNET) plus a first-degree family member with a confirmed MEN1 mutation (or if no genetic testing was performed); or (iii) two of the three major manifestations (including a dpNET) plus a first-degree family member meeting the same criteria. Exclusion criteria included any active non-NET malignancy, an active thymus NET or thymoma, rapidly progressive or metastatic lung or gastric NET, and poorly differentiated neuroendocrine carcinoma. Additional information is provided elsewhere [45].

#### Model training and testing in the real datasets

For the SCLC dataset, the initial split consisted of a development set with eight case-control matched pairs (D) and a testing set (T) with seven pairs. The development set was further divided into a development subset with five matched pairs (D1) and a validation subset with three matched pairs (D2). An additional validation set (D3) included three matched pairs randomly sampled from the D1.

For the MEN1 dataset, the dataset was randomly spited into 70% of development set (D; n = 39) and 30% of test set (T; n = 17). The development set was further divided into a training set (D1; n = 27) and validation set (D2; n = 12). An additional validation set (D3; n = 12) was randomly sample from D1 (Supplementary Fig. S7).

**Key Points**

- High-throughput omics data present major challenges for machine learning due to batch effects, missing values, and technical variability, often leading to reduced predictive accuracy.
- We introduce Rank-Based Learning (RBL), a novel method that leverages feature ranking to improve robustness against non-biological variations.
- RBL consistently outperforms Logistic Regression and Random Forest in simulations and in two plasma proteomics datasets for early-stage SCLC and dpNET detection.
- By improving prediction accuracy and generalizability, RBL offers a promising approach for developing more reliable diagnostic tools from omics data, with greater potential for clinical translation.

## Supplementary Material

Supplementary_figures_and_tables_bbaf666

Supplementary_Table_S7_bbaf666

Supplementary_Table_S8_bbaf666

## Data Availability

Simulated code resulting in generating simulation data this work is publicly available at: https://github.com/lulusong512/Rank-Based-Learning.git. Access to real-world clinical data can be provided upon reasonable request, subject to approval and completion of the required inter-institutional Material Transfer Agreement (MTA).
