# Supplementary material for: Rank-based learning: a novel high-throughput algorithm resilient to missing data and effective for datasets with small sample size
Source: Brief Bioinform. 2025 Dec 12;26(6):bbaf666. doi: 10.1093/bib/bbaf666 (PMC12914468; doi:10.1093/bib/bbaf666)
Supplement: Supplementary_figures_and_tables_bbaf666 [file supplementary_figures_and_tables_bbaf666.docx]

**Supplementary Document: Rank-Based Learning: A Novel High-Throughput Algorithm Resilient to Missing Data and Effective for Datasets with Small Sample Size**

*Lulu Song^1^, Hamid Khoshfekr Rudsari^1^, Johannes Fahrmann^2^, Jody Vykoukal^2^, Sam Hanash^2^, James P Long^1^, Kim-Anh Do^1^, Ehsan Irajizad^1^*

1- Department of Biostatistics, University of Texas MD Anderson Cancer Center

2- Department of Cancer Prevention, University of Texas MD Anderson Cancer Center

Corresponding author:

Ehsan Irajizad, [eirajizad@mdanderson.org](mailto:eirajizad@mdanderson.org)

**Table S1**. Performances of RBL and LR in simulation scenario 1 (n = 60, True Differential scenario)

| Percentage of true differential parameters (%) | 1 | 5 | 10 | 20 |
| --- | --- | --- | --- | --- |
| RBL Development AUC | 0.98 (0.98-0.99) | 1.00 (1.00-1.00) | 1.00 (1.00-1.00) | 1.00 (1.00-1.00) |
| RBL Test AUC | 0.82 (0.80-0.84) | 0.95 (0.94-0.96) | 0.99 (0.99-1.00) | 1.00 (1.00-1.00) |
| LR Development AUC | 0.94 (0.93-0.96) | 0.96 (0.94-0.97) | 0.97 (0.96-0.98) | 0.97 (0.96-0.98) |
| LR Test AUC | 0.83 (0.80-0.87) | 0.88 (0.86-0.91) | 0.85 (0.83-0.88) | 0.85 (0.83-0.88) |


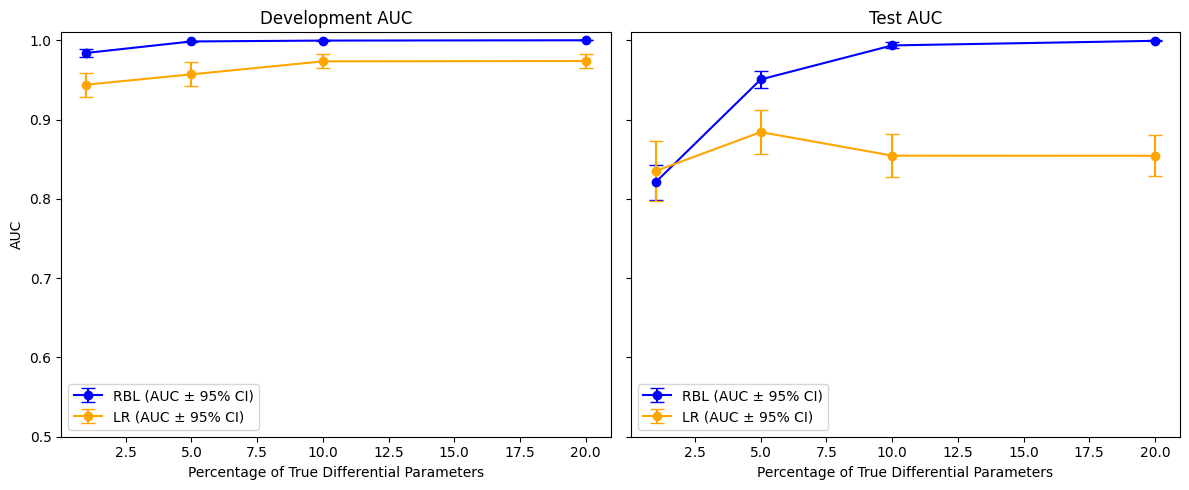


**Figure S1.** AUC performances of RBL and LR in simulation scenario 1 (n = 60, True Differential scenario)

**Table S2**. Performances of RBL and LR in simulation scenario 1 (n = 200, True Differential scenario)

| Percentage of true differential parameters (%) | 5 |
| --- | --- |
| RBL Development AUC | 0.98  (0.97-0.98) |
| RBL Test AUC | 0.90  (0.89-0.91) |
| LR Development AUC | 0.93  (0.91-0.94) |
| LR Test AUC | 0.85  (0.83-0.86) |

**Table S3.** Performances of RBL and LR in simulation scenario 2 (n = 60, Missing Data scenario)

| Percentage of Missing | 1% |  | 5% |  | 10% |  | 20% |  |
| --- | --- | --- | --- | --- | --- | --- | --- | --- |
| **RBL** | **train** | **test** | **train** | **test** | **train** | **test** | **train** | **test** |
| 10% | 0.98 | 0.81 | 1.00 | 0.93 | 1.00 | 0.99 | 1.00 | 1.00 |
|  | (0.98-0.99) | (0.79-0.83) | (0.99–1.00) | (0.92–0.95) | (1.00-1.00) | (0.99-1.00) | (1.00-1.00) | (1.00-1.00) |
| 20% | 0.98 | 0.77 | 0.99 | 0.91 | 1.00 | 0.98 | 1.00 | 0.99 |
|  | (0.97-0.98) | (0.74-0.79) | (0.99–1.00) | (0.90–0.93) | (1.00-1.00) | (0.98-0.99) | (1.00-1.00) | (0.99-1.00) |
| 30% | 0.97 | 0.76 | 0.99 | 0.88 | 1.00 | 0.97 | 1.00 | 0.99 |
|  | (0.97-0.98) | (0.73-0.78) | (0.99–1.00) | (0.86–0.90) | (1.00-1.00) | (0.97-0.98) | (1.00-1.00) | (0.98-0.99) |
| 40% | 0.97 | 0.72 | 0.99 | 0.87 | 1.00 | 0.95 | 1.00 | 0.98 |
|  | (0.96-0.98) | (0.70-0.75) | (0.99–0.99) | (0.85–0.89) | (1.00-1.00) | (0.94-0.96) | (1.00-1.00) | (0.98-0.99) |
| 50% | 0.97 | 0.72 | 0.99 | 0.82 | 0.99 | 0.92 | 1.00 | 0.95 |
|  | (0.96-0.97) | (0.69-0.74) | (0.98–0.99) | (0.80–0.84) | (0.99-1.00) | (0.90-0.94) | (1.00-1.00) | (0.94-0.97) |
| **LR** | **train** | **test** | **train** | **test** | **train** | **test** | **train** | **test** |
| 10% | 0.86 | 0.69 | 0.91 | 0.75 | 0.92 | 0.79 | 0.94 | 0.77 |
|  | (0.84-0.87) | (0.66-0.73) | (0.89-0.92) | (0.72-0.78) | (0.91-0.93) | (0.76-0.81) | (0.93-0.95) | (0.74-0.80) |
| 20% | 0.82 | 0.61 | 0.87 | 0.67 | 0.88 | 0.7 | 0.91 | 0.74 |
|  | (0.81-0.84) | (0.57-0.64) | (0.86-0.89) | (0.64-0.70) | (0.86-0.89) | (0.67-0.73) | (0.90-0.92) | (0.71-0.76) |
| 30% | 0.83 | 0.55 | 0.85 | 0.59 | 0.88 | 0.69 | 0.9 | 0.68 |
|  | (0.81-0.84) | (0.52-0.58) | (0.84-0.87) | (0.56-0.62) | (0.86-0.90) | (0.67-0.72) | (0.89-0.91) | (0.65-0.71) |
| 40% | 0.80 | 0.56 | 0.83 | 0.59 | 0.87 | 0.63 | 0.88 | 0.65 |
|  | (0.78-0.82) | (0.53-0.59) | (0.81-0.85) | (0.56-0.62) | (0.86-0.89) | (0.61-0.66) | (0.87-0.90) | (0.62-0.68) |
| 50% | 0.80 | 0.53 | 0.83 | 0.55 | 0.86 | 0.59 | 0.87 | 0.63 |
|  | (0.79-0.82) | (0.51-0.56) | (0.82-0.85) | (0.52-0.58) | (0.85-0.88) | (0.56-0.61) | (0.86-0.88) | (0.60-0.66) |


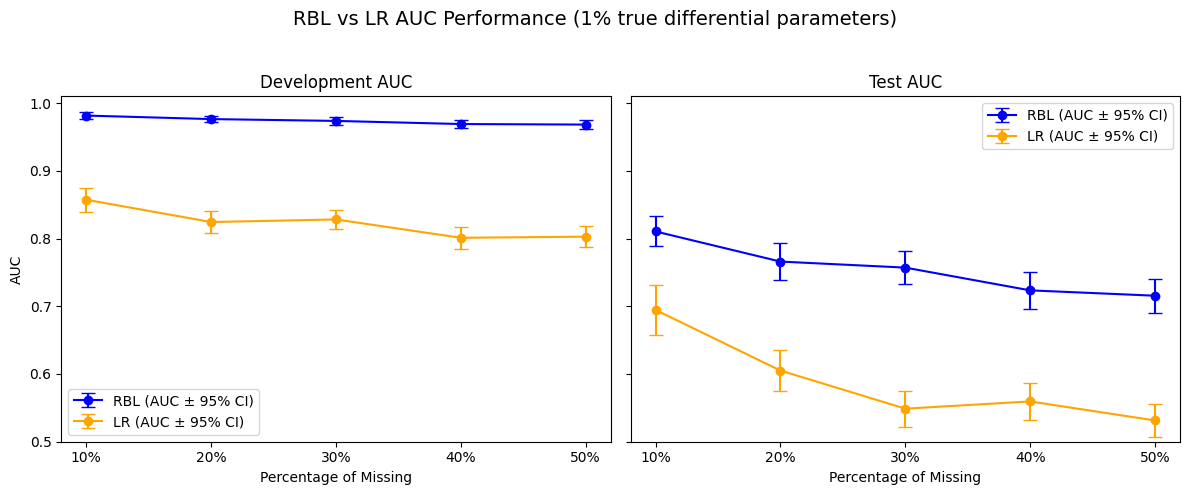


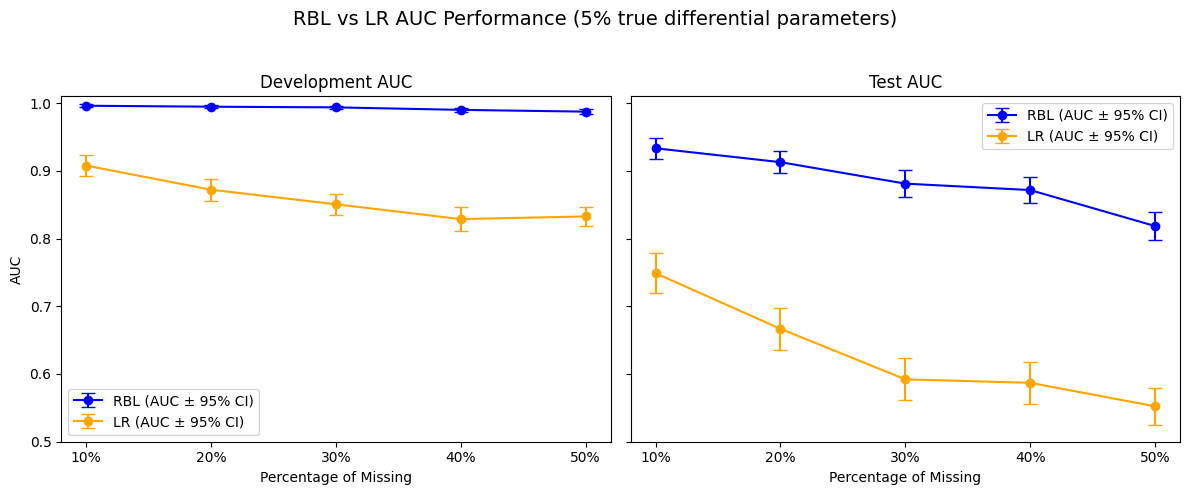


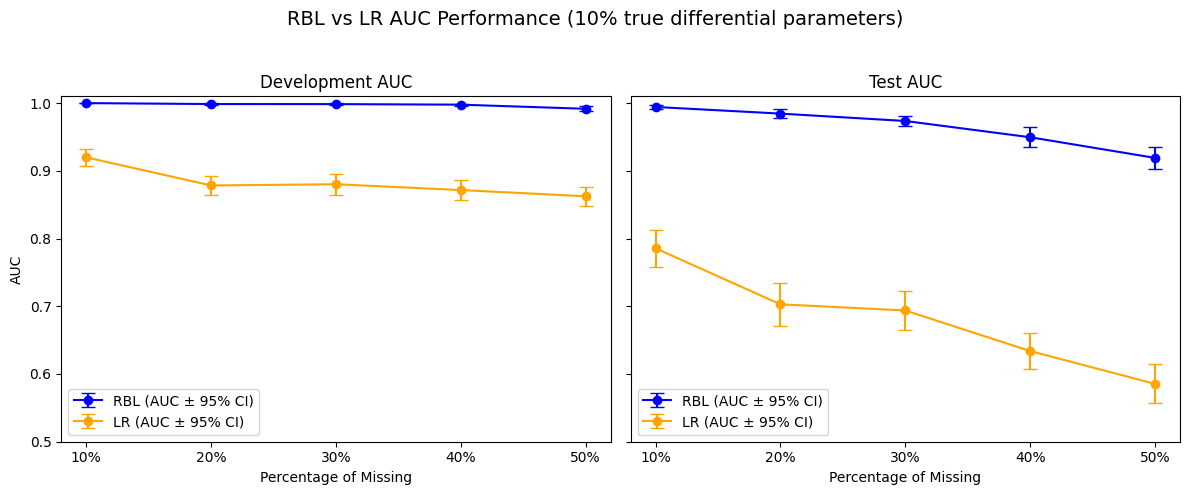


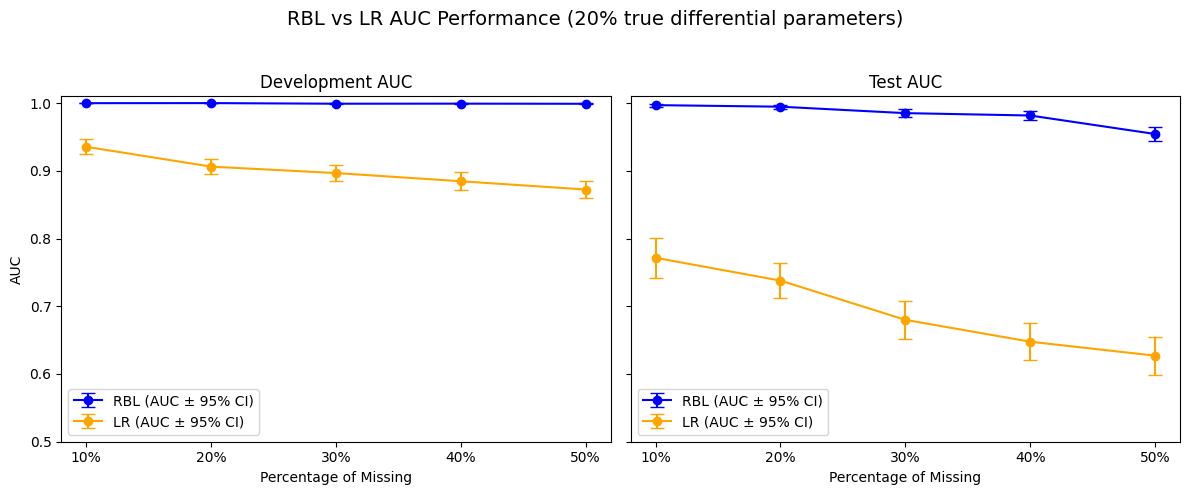


**Figure S2.** AUC performances of RBL and LR in simulation scenario 2 (n = 60, Missing Data scenario)

**Table S4**. Performances of RBL and LR in simulation scenario 3 (n = 60, Batch Effect scenario)

| Percentage of true differential parameters (%) | 1 | 5 | 10 | 20 |
| --- | --- | --- | --- | --- |
| RBL Development AUC | 0.98  (0.98-0.98) | 1.00  (1.00-1.00) | 1.00  (1.00-1.00) | 1.00  (1.00-1.00) |
| RBL Test AUC | 0.98  (0.98-0.98) | 1.00  (1.00-1.00) | 1.00  (1.00-1.00) | 1.00  (1.00-1.00) |
| LR Development AUC | 0.95  (0.94-0.96) | 0.97  (0.96-0.98) | 0.97  (0.95-0.98) | 0.97  (0.96-0.98) |
| LR Test AUC | 0.69  (0.67-0.70) | 0.70  (0.69-0.72) | 0.72  (0.70-0.73) | 0.71  (0.70-0.72) |


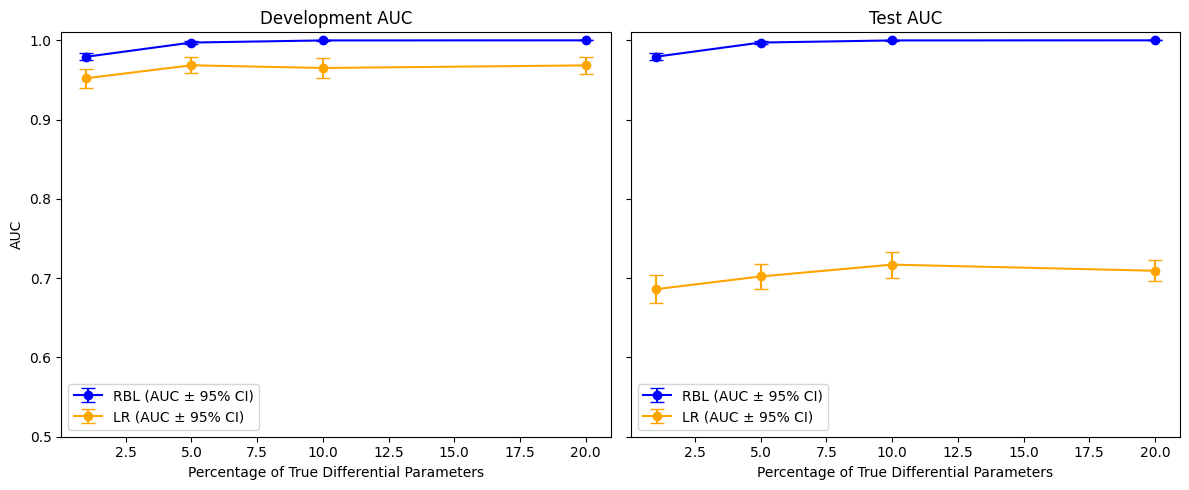


**Figure S3**. AUC performance of RBL and LR in simulation scenario 3 (n = 60, Batch Effect scenario)

| Percentage of true differential parameters (%) | 1 | 5 | 10 | 20 |
| --- | --- | --- | --- | --- |
| RBL Development AUC | 0.99 (0.99-0.99) | 1.00 (1.00-1.00) | 1.00 (1.00-1.00) | 1.00 (1.00-1.00) |
| RBL Test AUC | 0.86 (0.83-0.88) | 1.00 (0.99-1.00) | 1.00 (1.00-1.00) | 1.00 (1.00-1.00) |
| LR Development AUC | 0.98 (0.98-0.99) | 1.00 (1.00-1.00) | 1.00 (1.00-1.00) | 1.00 (1.00-1.00) |
| LR Test AUC | 0.96 (0.94-0.98) | 1.00 (1.00-1.00) | 1.00 (1.00-1.00) | 1.00 (1.00-1.00) |

**Table S5**. Performance of RBL and LR in simulation scenario 4 (n = 60, Correlation scenario)


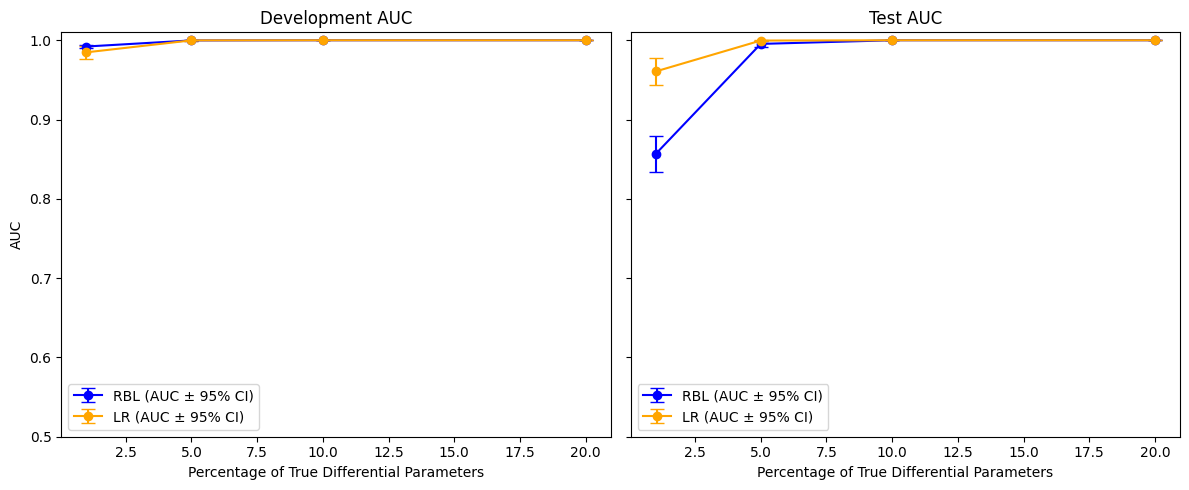


**Figure S4.** AUC performance of RBL and LR in simulation scenario 4 (n = 60, Correlation scenario)


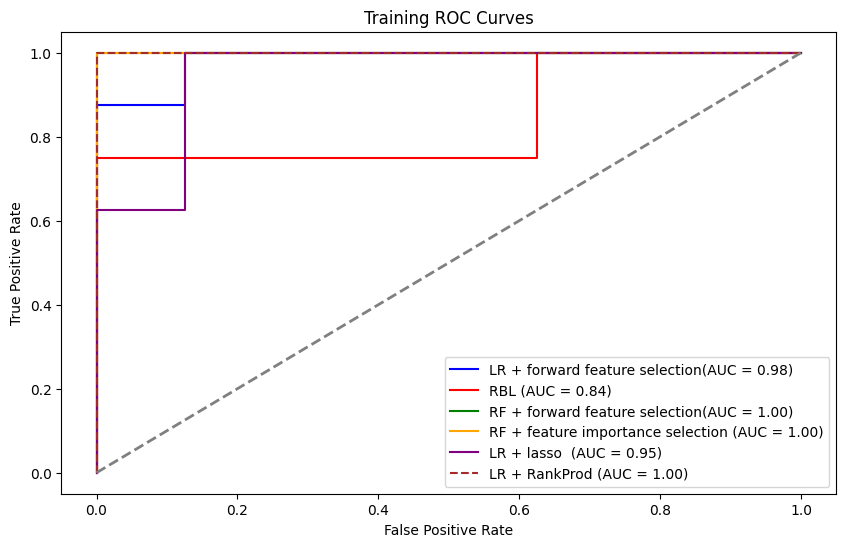


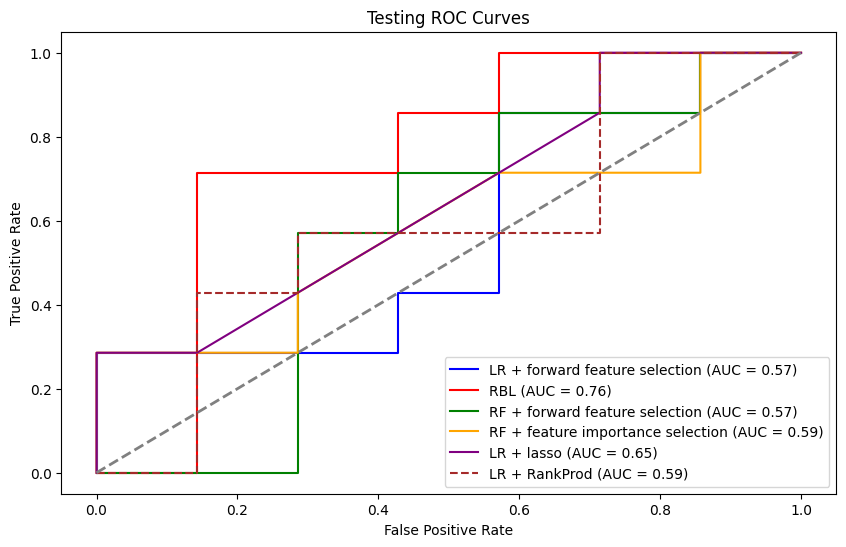


**Figure S5.** Performances of RBL, LR and LR Models for differentiating early-stage SCLC cancer from healthy controls


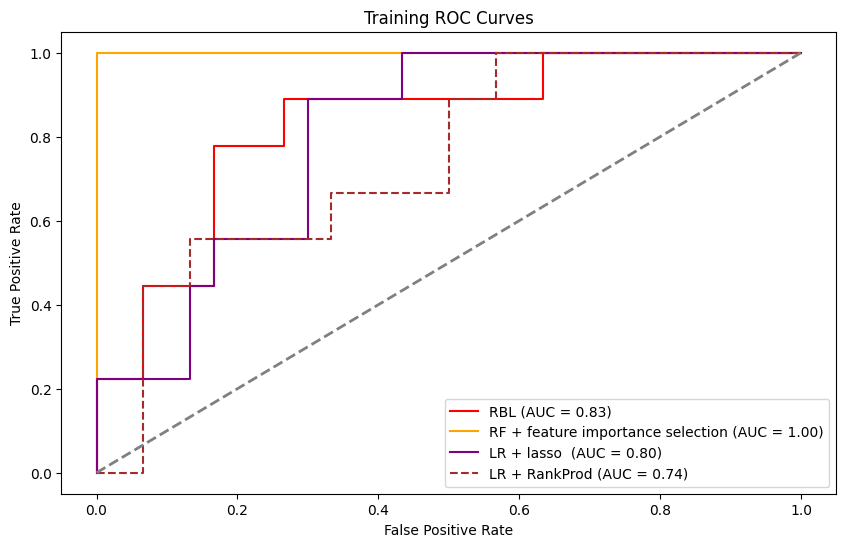


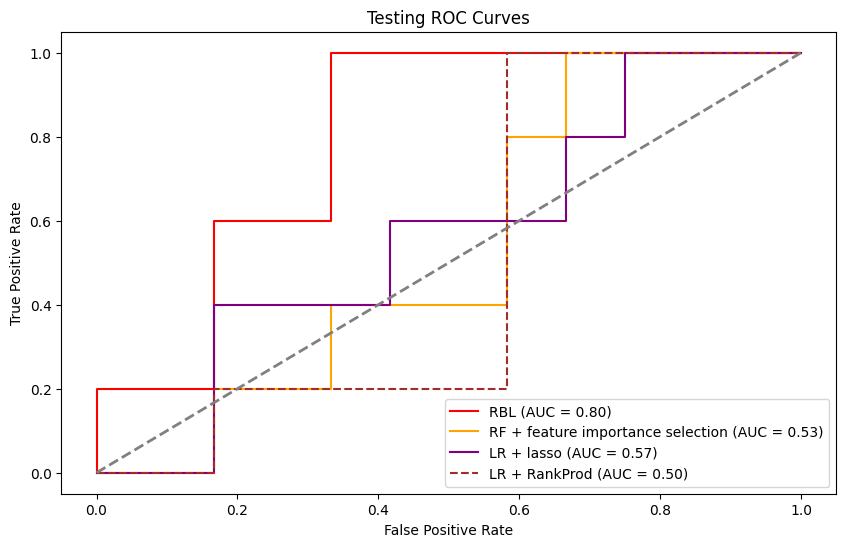


**Figure S6.** Performances of RBL, LR and LR Models for differentiating dpNET from controls with either a nonmetastatic indolent dpNET or no dpNET

**Table S6.** Computational settings and runtime of RBL across simulation and real data scenarios

| **Scenario** | **N**  **(sample)** | **K**  **(features)** | **C**  **(initialization runs)** | **T**  **(MCMC iterations)** | **Number of CPU** | **Average Runtime (second)** |
| --- | --- | --- | --- | --- | --- | --- |
| Base (simulation) | 60 | 300 | 100 | 600 | 12 | 66.8 |
| Base (simulation) | 200 | 300 | 100 | 603 | 12 | 234.4 |
| Missing (simulation) | 60 | 300 | 100 | 600 | 12 | 67.2 |
| Batch effect (simulation) | 120 | 300 | 100 | 600 | 12 | 75.7 |
| Correlation  (simulation) | 60 | 300 | 100 | 600 | 12 | 66.66 |
| Early-stage SCLC  (real data) | 30 | 4,390 | 100 | 600 | 12 | 3570.7 |
| MEN1  (real data) | 56 | 10,937 | 100 | 600 | 12 | 37754.46 |


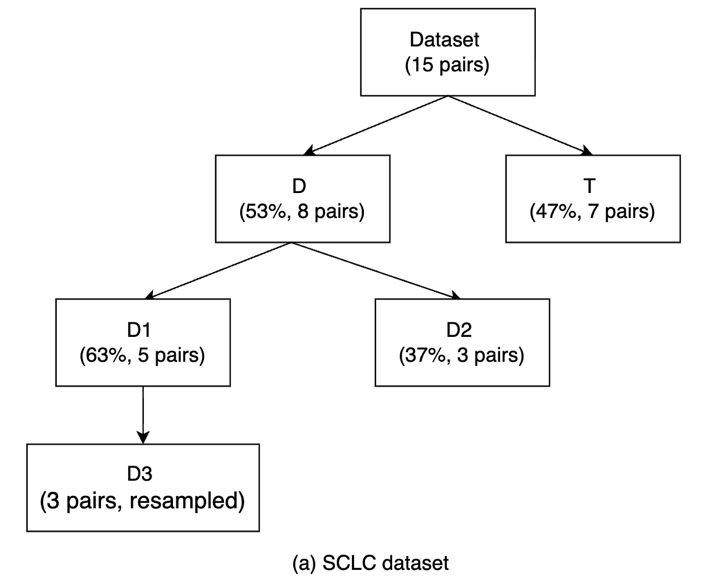

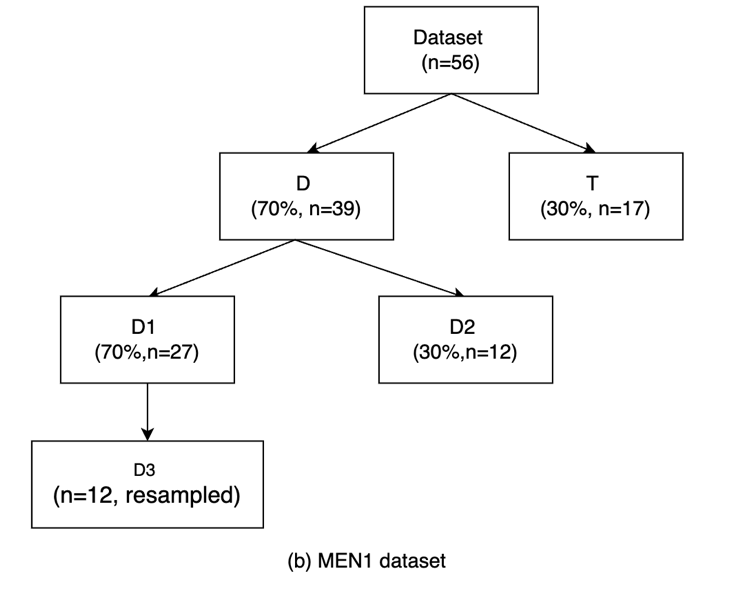


**Figure S7.** Data splitting strategy for model training, validation, and testing. (a) SCLC dataset: total 15 matched pairs, split into development (D) and test (T) sets.
D was further divided into D1 (training), D2 (validation), and D3 (resampled validation).
(b) MEN1 dataset: randomly divided into D (70%) and T (30%), then further split into D1 (training), D2 (validation), and D3 (resampled validation).
